# Impact of visiting restrictions on Edinburgh postnatal depression scale screening scores at one month postpartum during the spread of COVID-19: a single-center case-control study in Japan

**DOI:** 10.1186/s12884-023-05979-7

**Published:** 2023-09-09

**Authors:** Sho Kudo, Harumichi Banno, Taro Itou, Hiroshi Kawamura, Daisuke Inoue, Nozomu Takahashi, Makoto Orisaka, Yoshio Yoshida

**Affiliations:** 1 Department of Obstetrics and Gynecology, Maizuru Kyosai Hospital, Hama, Maizuru-shi, Kyoto, 1035, 625-0036 Japan; 2 Faculty of Medical Sciences, Department of Obstetrics and Gynecology, University of Fukui, 23-3 Matsuokashimoaiduki, Yoshida-gun Eiheiji-cho, Fukui, 910-1104 Japan

**Keywords:** COVID-19, Visiting restrictions, Edinburgh postnatal depression scale, Postpartum depression

## Abstract

**Background:**

This study aimed to evaluate whether “visiting restrictions” implemented due to the coronavirus disease 2019 (COVID-19) pandemic are a risk factor for postpartum depression using the Edinburgh Postnatal Depression Scale (EPDS).

**Methods:**

This case-control study participants who gave birth during the spread of COVID-19 (COVID-19 study group) and before the spread of COVID-19 (control group). Participants completed the EPDS at 2 weeks and 1 month after childbirth.

**Results:**

A total of 400 cases (200 in each group) were included in this study. The EPDS positivity rate was significantly lower with visiting restrictions than without (8.5% vs.18.5%, p = 0.002). Multivariate analysis of positive EPDS screening at the 1st month checkup as the objective variable revealed that visiting restrictions (odds ratio (OR): 0.35, 95% confidence interval (CI): 0.18–0.68), neonatal hospitalization (OR: 2.17, 95% CI: 1.08–4.35), and prolonged delivery (OR: 2.87, 95% CI: 1.20–6.85) were factors associated with an increased risk of positive EPDS screening.

**Conclusion:**

Visiting restrictions on family during the hospitalization period for delivery during the spread of COVID-19 pandemic did not worsen EPDS screening scores 1 month postpartum, but stabilized the mental state of some mothers.

## Background

The mental health of pregnant and nursing mothers associated with the coronavirus disease 2019 (COVID-19**)** pandemic has become a serious concern. A recent survey showed that many pregnant women felt anxious about not receiving the needed support due to the “prohibition on visits and overnight stays during hospitalization” and “prohibition on witnessing births” because many medical facilities prohibited visits and overnight stays during hospitalization during the COVID-19 pandemic [1]. However, some expectant mothers reported that the lack of visitors allowed them to rest and reduced stress, and that facing the child after birth was advantageous for attachment formation [1]. Additionally, others reported that they received more support from their husbands than before because they spent more time at home due to their husbands increased telecommuting hours due to the COVID-19 pandemic [1].

Postpartum depression (PPD) is a mental disorder that can occur after childbirth [2]. The incidence of PPD is approximately 17.2% worldwide and 14.3% in Japan [3, 4]. In contrast to maternity blues, PPD persists for more than 2 weeks and is not temporary. PPD is associated with adverse effects on the child’s cognitive, emotional, social, and behavioral development in the short and long- term [5–7]. Furthermore, PPD can lead to maternal suicide, clearly representing severe problems. A previous study in Taiwan compared mothers who experienced childbirth with PPD and those who experienced childbirth without PPD and reported a hazard ratio of suicide among mothers with PPD of 19.3 [8]. The PPD etiology is not clearly identified, but it is presumed by some factors, such as neuroendocrine changes, neuroinflammation, neurotransmitter alterations, circuit dysfunction, and the involvement of genetics and epigenetics [9]. The main treatment options for PPD include drug therapy, such as selective serotonin reuptake inhibitors, and psychotherapy, which may require a long period of time [2]. Some studies reported that the economic burden PPD is approximately 74,000£ per case [10]. Another study reported that children of mothers with PPD incurred 12% higher total healthcare costs in the first 24 months of life than children of mothers without PPD [11]. Therefore, efforts should be made to identify important risk factors for PPD and to implement appropriate countermeasures. Previous studies have reported that economic hardship, lack of partner support, unwanted (or unintended) pregnancy, and a history of psychiatric illness are risk factors associated with PPD [12–16]. However, no study has reported the impact of visiting restrictions during and after delivery on the mental health of postpartum mothers, as no one has experienced a worldwide pandemic like COVID-19 before.

In Japan, family visits during delivery are considered a positive thing and are prominently promoted through well-advertised television or parenting magazines. The Japanese Ministry of Health reported that 86% of mothers stay with their family during delivery, and the percentage of witnessed births is higher than before [17].Additionally, the Cochrane Library reported that continuous support from family plays an important role in reducing negative feelings about childbirth [18]. Moreover, some studies have shown that the low level of satisfaction with delivery worsens depression symptoms after birth [19, 20]. Based on these studies, social perception, and our experience, we hypothesized that visiting restrictions during the COVID-19 pandemic would worsen the results of the Edinburgh Postnatal Depression Scale (EPDS), which is a screening tool for PPD, and the EPDS positivity rate would increase after visiting restrictions. The hypothesis was directional.

This study aimed to evaluate whether “prohibition on visits and overnight stays during hospitalization due to delivery” and “prohibition on witnessed births”, as implemented by many medical institutions in the context of the spread of COVID-19, increase depressive symptoms during pregnancy using the EPDS.

## Methods

### Participants

This was a case-control study. Mothers who gave birth from April 3, 2019, to December 16, 2020, at Maizuru Kyosai Hospital were included in this study. Exclusion criteria includes mothers missing EPDS scores at 1st month or those with stillborn babies because it would be natural that mothers who experienced stillbirth would be depressed. This study was approved by the Ethics Committee of Maizuru Kyosai Hospital (Approval no. 2,022,002). All mothers who were screened positive on the EPDS were referred to a doctor at our hospital. If the doctor regarded that a medical examination by a psychiatrist was necessary, the mothers were referred to a specialized hospital. The participants were divided into two groups (control and COVID-19 study groups). The control group included women admitted to the Maternity Center of Maizuru Kyosai Hospital from April 3, 2019, to February 29, 2020, where families and friends were allowed to visit mothers freely in the day room of the ward during the period of hospitalization for delivery. The COVID-19 study group included women admitted from March 1, 2020, to December 16, 2020, where all visits were prohibited from admission time until discharge due to theCOVID-19 pandemic.

### Procedure

Data on delivery status, including the presence or absence of neonatal hospitalization, delivery time, blood loss, and history of psychiatric illness, and EPDS screening at the 1st month checkup were retrospectively collected from the medical records of 200 pregnant women who delivered a live baby (the COVID-19 study group). Cases with no EPDS entry in the medical record for the first postpartum month (152 before restriction and 48 after restriction) and 21 cases who delivered between July 1 and July 21, 2020, when the temporary visiting restrictions were lifted due to improvements in the COVID-19 situation, were excluded from this study. In 69 participants, EPDS scores at the 2nd week checkup were missing.

The self-administered EPDS at the 2nd week and 1st month checkups were collected in person, with the EPDS at 1st month being used as the primary endpoint. The EPDS includes 10 items. Each is rated on a 4-point scale from 0 to 3. The total score ranges from 0 to 30. This questionnaire was developed by John Cox in 1987 [21]. The EPDS is a commonly used to identify women who may have PPD. The sensitivity and specificity of the EPDS vary slightly by language. In 1997, Okano et al. translated EPDS into Japanese [22] and tested the EPDS reliability in Japanese. In their study 47 postpartum mothers were included, and the EPDS data were collected at 1 and 3 months after delivery and compared with those of the control group to predict depression symptoms. They reported that the sensitivity and specificity of prediction in the Japanese version were 0.75 and 0.93, respectively [22]. The Consensus Guide for Perinatal Mental Health in Japan 2017 recommends screening all mothers using the postpartum EPDS to identify mothers at high risk for PPD and allow appropriate action [23]. According to the criteria of Okano et al., a score of ≥9 was considered a positive result for EPDS screening [22].

The time required for delivery was defined as the time from the onset of labor pain to the delivery of the placenta, excluding cases of scheduled cesarean section. According to the definitions of the Japanese Society of Obstetrics and Gynecology, prolonged delivery was defined as ≥30 h for first-time mothers and ≥15 h for multipara [24]. The blood loss amount was calculated based on measurements taken up to 2 h after delivery. Neonatal hospitalization was defined as hospitalization for at least 1 day before the mother was discharged from the hospital (day 6 postpartum for first-time mothers; and day 5 postpartum for postpartum mothers). Cases with a history of psychiatric visits were defined as those with a history of psychiatric illness.

### Statistical analyses

All statistical analyses were performed using Excel Statistics version 3.23 (Bell Curve, Tokyo, Japan).

The Shapiro-Wilk test was conducted on each numerical data. the results indicated that all data were non-normally distributed. The data were presented as median. Differences in the distribution of categorical variables were examined using the Mann–Whitney U and χ^2^ tests. Logistic regression analysis was performed to analyze the relationship between the independent and dependent variables. Explanatory variables were selected based on previous research and data (Table 1) [12–16]. A p- value of < 0.05 was considered significant.

A complete case analysis was performed in all analyses.

## Results

A total of 200 women who applied for delivery before the visiting restriction period and 200 women who applied during the restriction period were included in the study. Table 1 shows the characteristics of the study participants. No statistical differences in maternal age, gravida, parity, weeks of delivery, and delivery method were observed between the two groups. The control group showed a shorter duration of labor and less blood loss. The number of patients with a history of psychiatric illness was lower with visiting restrictions (13% vs. 2%, p < 0.001). No significant differences in other factors were observed.

**Table 1:** Characteristics of the study participants

	Without visiting restrictions	With visiting restrictions	p
Maternal age (years)	31 [18-45]	31 [19-43]	0.638
Primipara	77 (38.5%)	80 (40.0%)	0.759
Multipara	123 (61.5%)	120 (60.0%)	
Weeks of delivery	39 [30-41]	39 [33-42]	0.109
Birth weight (g)	2996 [1277-4070]	3014 [1822-4165]	0.791
Delivery method			
Vaginal delivery	189 (94.5%)	190 (95.0%)	0.823
Planned cesarean section	6 (3.0%)	3 (1.5%)	0.312
Emergency cesarean section	5 (2.5%)	7 (3.5%)	0.558
Duration of labor (min)	405	454.5	0.006
Blood loss (g)	439 [88-3934]	541 [70-3660]	0.021
History of psychiatric illness			
With	26	4	
Without	174	196	
Rate of psychiatric illness	13%	2%	< 0.001

Categorical variables are presented as numbers (%), and continuous variables are presented as median [range]. Numerical variable were compared using the Mann-Whitney U test, and categorical variables were compared using the chi-square test.

Table 2 shows the results of the comparisons of percentages of patients with positive EPDS screening and median EPDS scores in the control and COVID-19 study groups.

**Table 2:** Comparison of EPDS scores and positivity rate

	Without visiting restrictions	With visiting restrictions	p
Number of deliveries	200	200	
EPDS 2 weeks (n = 317)	143	174	
EPDS < 9 (n = 266)	115 (80.4%)	151 (86.8%)	
EPDS ≥ 9 (n = 51)	28 (19.6%)	23 (13.2%)	0.141
Positivity rate	19.58%	13.22%	0.141
Median EPDS	4.0 [0-23]	3.0 [0-19]	0.337
EPDS 1 month (n = 400)	200	200	
EPDS < 9 (n = 346)	163 (81.5%)	183 (91.5%)	
EPDS ≥ 9 (n = 54)	37 (18.5%)	17 (8.5%)	0.0034
Positivity rate	18.5%	8.5%	0.0034
Median EPDS	3.0 [0-22]	3.0 [0-18]	0.091

EPDS, Edinburgh Postnatal Depression Scale

Continuous variables are presented as median[range]

Numerical variable were compared using the Mann-Whitney U test, and categorical variables were compared using the chi-square test was used to compare categorical variable.

No significant differences in the EPDS positivity rate (19.58% vs. 13.22%, p = 0.141) or median EPDS score (4.0 vs. 3.0, p = 0.337) were observed at the 2nd week checkup. However, the values for both tended to be lower in the COVID-19 study group. The EPDS positivity rate was significantly lower in the COVID-19 study group at the 1st month checkup. (18.50% vs. 8.50%, p = 0.0034).

In the sensitivity analyses, the results remained significant for all mothers except those with history of psychiatric illness(17.80% vs. 8.10%, p = 0.0051).

A significant difference in the EPDS positivity rate was observed between the COVID-19 study group and the Control group (8.5% vs. 18.5%, p = 0.002).

Table 3 shows the results of the comparison of screening-positive and -negative patients.

**Table 3:** Comparison of the delivery situation and medical history by EPDS score

	< 9 (*n* = 346)	≥9 (*n* = 54)	p
Maternal age	31.0 [18-45]	33.0 [20-42]	0.134
Primipara	129	28	
Multipara	217	26	
Weeks of delivery	39.0 [30-42]	39.0 [36-42]	0.086
Birth weight (g)	3010.0 [1297-4165)	3025.0 [2096-4055)	0.391
Delivery method			
Vaginal delivery	328 (94.8%)	51 (94.4%)	0.914
Planned cesarean section	9 (2.6%)	0 (0%)	
Emergency cesarean section	9 (2.6%)	3 (5.6%)	
Duration of labor (min)	438 [49-3877)	415 [85-4457]	0.642
Blood loss (g)	481 [79-3934]	510.5 [190-2254]	0.354
History of psychiatric illness			
With (*n* = 30)	23 (76.7%)	7 (23.3%)	0.101
Without (*n* = 370)	323 (87.3%)	47 (12.7%)	
Visiting restrictions			
With (*n* = 200)	183 (91.5%)	17 (8.5%)	0.002
Without (*n* = 200)	163 (81.5%)	37 (18.5%)	

Continuous variables are presented as median [range]

Numerical variables were compared using the Mann-Whitney U test, and categorical variables were compared using the chi-square test.

Table 4 shows the results of multivariate analysis using positive EPDS screening at the 2nd week checkup as the objective variable.

The analysis revealed that previous experience of delivery (odd ratio(OR) 0.29, p = 0.001) and history of psychiatric illness(OR 4.68, p = 0.001) were predictive factors.

**Table 4:** Resilience factors predicting EPDS positivity at 2nd weeks postpartum

Variables	OR (95% CI)	p
Multipara	0.29 (0.15–0.59)	0.001
Restricted visits	0.74 (0.38–1.46)	0.389
Cesarean section	0.91 (0.09–8.71)	0.932
History of psychiatric illness	4.68 (1.83–11.99)	0.001
Presence of neonatal hospitalization	1.19 (0.52–2.75)	0.667
Prolonged delivery	1.32 (0.52–3.33)	0.558

OR, odds ratio; CI, confidence interval

OR, was calculated by adjusting positive EPDS screening at the 2-week checkup for primiparous status, presence of restricted visits, type of delivery, history of psychiatric illness, presence of neonatal hospitalization, and presence of prolonged delivery

Table 5 shows the multivariate analysis results of the EPDS scores 1st month after delivery. This analysis showed that visiting restrictions (OR 0.35, p = 0.002), neonatal hospitalization (OR 2.17, p = 0.030), and prolonged delivery (OR 2.87, p = 0.017) were factors affecting the EPDS positivity rate.

**Table 5:** Resilience factors predicting EPDS positivity 1st month after delivery

Variables	OR (95% CI)	p
Multipara	0.78 (0.41–1.48)	0.439
Restricted visits	0.35 (0.18–0.68)	0.002
Cesarean section	3.05 (0.48–19.19)	0.235
History of psychiatric illness	1.73 (0.66–4.55)	0.265
Neonatal hospitalization	2.17 (1.08–4.35)	0.030
Prolonged delivery	2.87 (1.20–6.85)	0.017

OR, odds ratio; CI, confidence interval

OR was calculated by adjusting positive EPDS screening at the 1st month checkup for primiparous status, presence of restricted visits, type of delivery, history of psychiatric illness, presence of neonatal hospitalization, and presence of prolonged delivery

## Discussion

In this study, visiting restrictions on family members during hospitalization for delivery during the COVID-19 pandemic did not worsen the EPDS positivity rate 1st month postpartum, this significantly reduces the risk of EPDS by 65%. Indeed, restrictions contributed to the stabilization of the mental state of the mother. Many studies have estimated the impact on maternal mental health. However, there is no study has focused on visiting restrictions. Therefore, this study is meaningful for us and suggests how to maintain the delivery system during a pandemic.

Prolonged delivery, absence of visiting restrictions, and early hospitalization of the neonate had the a significant effect on EPDS screening values at the 1st month checkup.

Several studies have examined postpartum mental health during the COVID-19 pandemic. Gluska et al. examined the incidence of PPD using the EPDS screening in 420 women [25]. In their study, fear of the COVID-19 pandemic was identified as a risk factor for PPD during the COVID-19 pandemic (OR = 1.11). Mollard et al. in their study on 885 pregnant women using the Perceived Stress Scale (PSS-10). reported increased maternal stress during the COVID-19 pandemic, particularly when the infant was admitted to the neonatal intensive care unit. They also stated that stable income and employment status reduced stress [26] Furthermore, Hui et al. reported an increase in the proportion of pregnant women who were EPDS-positive after the COVID-19 pandemic in a retrospective study of 4531 pregnant women before and after the COVID-19 pandemic (before, 11.9% vs. after, 14.4%, p < 0.05) [27]. In 2022, Safi-Keykaleh et al. conducted a meta-analysis of observational studies to examine the prevalence of PPD in both Persian and English women during the COVID-19 pandemic. The results indicated an increase in PPD due to the COVID-19 pandemic. However, this analysis showed very high heterogeneity between studies due to significant differences in social backgrounds (e.g., religion, and economic status), sample size, tools used, and cut-off values [28].

Pariente et al. compared the EPDS positivity rate of 346 individuals before and after the COVID-19 pandemic. They reported a predominant decrease in positivity after the COVID-19 pandemic (before, 6.8% vs. after, 15.2%, p = 0.014) [29].

Previous reports have claimed increases and decreases in maternal mental health during the COVID-19 pandemic, depending on the region. The common thread among these reports is that family support and financial stability reduce stress. Similar findings were reported in studies before the COVID-19 pandemic [30].

In this study, contrary to prior expectations, visiting restrictions for a short time before and after delivery during the COVID-19 pandemic did not worsen the EPDS positivity rate. This may be because visiting restrictions may have eliminated the need for unwanted visits and promoted postpartum recovery from fatigue. The COVID-19 pandemic reduced the frequency of going out and eating out, and the promotion of remote work reduced burdens on the mother by encouraging partners to spend more time at home and cooperate in childcare. Additionally, compared with other countries, Japan’s stable economy and relatively controlled spread of infection may have reduced the mothers’ mental stress.

The EPDS positivity rate at the 2nd week checkup was higher among first-time mothers and mothers with a history of mental illness, regardless of the presence or absence of restrictions on visitation. These findings suggest that first-time mothers should concentrate on learning childcare skills and adapting to the environment immediately after delivery. It is important to carefully manage mothers with a history of psychiatric illness. Furthermore, early hospitalization of infants after birth worsened EPDS screening values. In this study, delivery method did not affect postpartum EPDS screening values, but an association was observed between prolonged delivery and an increased EPDS positivity rate. These findings suggest that appropriate interventions at the right time, regardless of the delivery method, can reduce maternal fatigue and positively affect postpartum mental status. The study results revealed that infection control might be prioritized over continuing family visits during birth during nationwide pandemic. This study has several limitations. First, this was a single-center, retrospective study. The facility where this study was conducted has a policy of minimal medical interventions in managing labor and delivery. Additionally, the ratio of cesarean sections to the total number of deliveries is extremely low compared with that of a general hospital. Different trends can be found in facilities with higher cesarean section rates. Second, not only medical factors but also social factors, such as being a single parent or not, household income, partner’s job, and living with grandparents or not, can affect EPDS scores. Third, we only retrospectively checked the EPDS scores using medical records. We did not check the data, apart from medical data, such as the mother’s job, income, and family structure. Furthermore, a difference in the ratio of pregnant women with a history of psychiatric illness with or without visiting restrictions was observed, which may represent a confounding factor. Additionally, the relationship between pregnant women and their partners’ mothers is special in Japanese culture. If the partners’ mothers want to see their grandchildren, pregnant women cannot reject their visitation. Finally, because the EPDS is only a screening tool, positive EPDS screening does not directly lead to diagnosing of PPD. In this study, the outcomes after EPDS screening were not followed. Further studies are needed to verify this point and examine whether patients with positive EPDS results are subsequently diagnosed with PPD.

Despite these limitations, this study provides suggestive evidence on maintaining the delivery system during a pandemic. Evidence prioritizing infection control over family visits during birth was lacking before this study.

## Conclusion

This study showed that visiting restrictions imposed during COVID-19 pandemic did not increase the risk for PPD symptoms, on the contrary, this significantly reduces the risk of EPDS by 65%. During an explosive outbreak of an infectious disease, greater merits may be seen in prioritizing nosocomial infection prevention and imposing strict infection control measures rather than maintaining a system that allows visitation out of consideration for the mental health of pregnant women. Additionally, offering the option of prioritizing rest over visitation to pregnant women with accumulated fatigue or a history of mental illness may be desirable regardless of the presence or absence of infectious disease outbreaks. Furthermore, avoiding prolonged delivery and hospitalization of the newborn through appropriate medical intervention, including cesarean section, would be desirable to reduce PPD.

## Data Availability

The datasets used and analyzed during this study are available from the corresponding author upon reasonable request.

## Abbreviations

COVID-19: Coronavirus disease 2019
EPDS: Edinburgh Postnatal Depression Scale
PPD: Postpartum depression
